# Endogenous fluctuations in the dopaminergic midbrain drive behavioral choice variability

**DOI:** 10.1073/pnas.1900872116

**Published:** 2019-08-26

**Authors:** Benjamin Chew, Tobias U. Hauser, Marina Papoutsi, Joerg Magerkurth, Raymond J. Dolan, Robb B. Rutledge

**Affiliations:** ^a^Max Planck University College London Centre for Computational Psychiatry and Ageing Research, London WC1B 5EH, United Kingdom;; ^b^Wellcome Centre for Human Neuroimaging, University College London, London WC1N 3BG, United Kingdom;; ^c^Huntington’s Disease Centre, University College London, London WC1B 5EH, United Kingdom

**Keywords:** behavioral variability, intrinsic brain fluctuations, dopaminergic midbrain, risky decision making, real-time fMRI

## Abstract

Human behavior is surprisingly variable, even when facing the same problem under identical circumstances. A prominent example is risky decision making. Economic theories struggle to explain why humans are so inconsistent. Resting-state studies suggest that ongoing endogenous fluctuations in brain activity can influence low-level perceptual and motor processes, but it remains unknown whether endogenous fluctuations also influence high-level cognitive processes including decision making. Here, using real-time functional magnetic resonance imaging, we tested whether risky decision making is influenced by endogenous fluctuations in blood oxygenation level-dependent (BOLD) activity in the dopaminergic midbrain, encompassing ventral tegmental area and substantia nigra. We show that low prestimulus brain activity leads to increased risky choice in humans. Using computational modeling, we show that increased risk taking is explained by enhanced phasic responses to offers in a decision network. Our findings demonstrate that endogenous brain activity provides a physiological basis for variability in complex human behavior.

## Significance

Humans are surprisingly inconsistent in their behavior, often making different choices under identical conditions. Previous research suggests that intrinsic fluctuations in brain activity can influence low-level processes, such as the amount of force applied in a motor response. Here, we show that intrinsic prestimulus brain activity in the dopaminergic midbrain influences how we choose between risky and safe options. Using computational modeling, we demonstrate that endogenous fluctuations alter phasic responses in a decision network and thereby modulate risk taking. Our findings demonstrate that higher-order cognition is influenced by fluctuations in internal brain states, providing a physiological basis for variability in complex human behavior.

Human behavior is inherently variable. Even when facing the same task repeatedly, humans often act in inconsistent ways. This observation led the English poet Horace Smith to suggest that “inconsistency is the only thing in which men are consistent.” Inconsistencies in value-based decision making often violate the tenets of rational economic theory. Many economic models explain this variability by injecting stochasticity into subjective preferences (1).

The human brain shows substantial regional activity fluctuations in the absence of external stimulation (i.e., resting state) (2, 3). The functional role of these fluctuations is not well understood. Endogenous fluctuations endure when participants perform externally imposed tasks and can explain neural variability in task-evoked responses (4). Studies investigating low-level cognitive processes have shown that endogenous fluctuations also influence how stimuli are processed. Endogenous fluctuations in task-relevant areas influence perception of auditory (5) and somatosensory stimuli (6) and can influence the force exerted during simple motor actions, such as button presses (7). However, it remains unknown whether intrinsic fluctuations also affect complex cognitive processes, such as decision making, and whether variability in prestimulus brain activity can predict future decisions.

In this study, we hypothesized that endogenous fluctuations in areas implicated in decision making would explain variability in choice. In particular, we hypothesized that endogenous fluctuations in the dopaminergic midbrain, encompassing substantia nigra and ventral tegmental area (SN/VTA), play a key role in decision making under risk. SN/VTA contains the largest assembly of dopamine neurons in the human brain and is centrally involved in decision making (8, 9). Modulating dopamine neurotransmission can increase risk taking (10–12), and dopamine dysfunction is strongly linked to problem gambling and impulsive behaviors (13). Although it is not possible to directly assess dopaminergic activity using functional MRI (fMRI), dopamine-related quantities such as reward prediction errors (14) are observed in BOLD activity within the SN/VTA (15, 16).

**Fig. 1.**
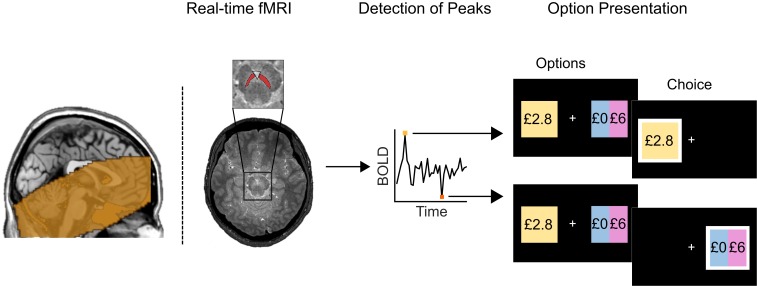
Schematic of real-time fMRI setup. BOLD activity from anatomically defined SN/VTA is extracted and denoised (removing movement, breathing, and pulsatile artifacts) in real time. The overlay on the sagittal image indicates intersecting coverage across all subjects in the study. Endogenous activity reflecting a low/high background activity state (exceeding a 15th/85th percentile cutoff) triggered presentation of a trial with a choice between a safe option (here, £2.8 guaranteed reward) and a risky option (here, £0 or £6 with equal probability). To ensure similar rates of risk taking across individuals, safe options varied around each subject’s indifference point, which was determined prior to scanning. This design allowed us to efficiently and selectively probe subjects with identical options during very low and very high endogenous SN/VTA activity. Any difference in behavior can therefore be attributed to endogenous SN/VTA activity.

To test our hypothesis, we developed a real-time fMRI framework to trigger presentation of options based on intrinsic fluctuations of BOLD activity (17, 18) (Fig. 1 and *SI Appendix*, Fig. S1). We developed an algorithm that detected epochs of very high and very low activity, providing a trigger to probe subjects with a matched set of choices between a safe and a risky option in these 2 background brain states (*Methods*) (11, 12). The risky option comprised equal probabilities of a prize (£6, £9, or £12) or £0. The value of the safe option was always lower than the potential prize from the risky option and varied systematically around each subject’s economic indifference point, the offer for which a subject chooses safe and risky options in equal proportion. Safe option values were determined from prescanning decisions from an extensive choice set (*Methods*).

## Results

### Endogenous Fluctuations in SN/VTA BOLD Activity Modulate Risk Taking.

**Fig. 2.**
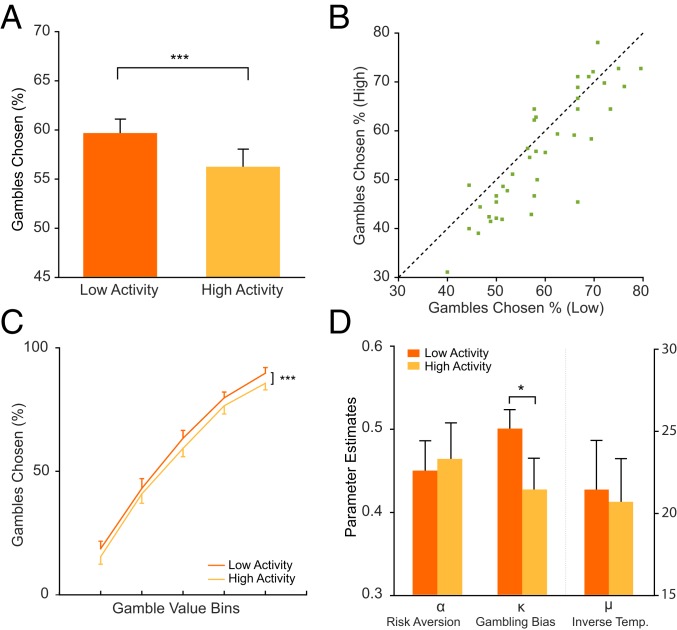
Endogenous fluctuations in SN/VTA BOLD activity modulate value-independent influences on choice. (*A*) Subjects (*n* = 43) gambled more when options were presented against a background of low compared to high endogenous SN/VTA activity. (*B*) This effect of greater risk taking for low than high activity was consistent across subjects. (*C*) The activity-induced shift in risk taking was independent of value with low endogenous activity leading to increased risk taking irrespective of option value. Differences in objective value between risky and safe options were divided into bins of equal sizes for each subject. (*D*) Choices were fitted to a parametric decision model based on prospect theory with the best-fitting model including a gambling bias parameter that was higher when endogenous activity was low. Positive gambling bias parameters reflects a tendency to take risks irrespective of option value. *P < 0.05 and ***P < 0.001. Data are mean ± SEM.

We first asked how the 2 modes of endogenous SN/VTA activity (low and high) influenced choice behavior. On average, subjects chose the risky option more when prestimulus SN/VTA activity was low compared to when it was high [low activity, 59.6 ± 1.5% (mean ± SEM); high activity, 56.2 ± 1.8%; *t*_(42)_ = 3.83, *P* < 0.001; Fig. 2*A*]. This effect of greater risk taking following low compared to high activity was present in 30 of 43 subjects (Fig. 2*B*). Post hoc off-line control analyses suggest that this relationship is unaffected by the precise timing of option presentation or degree of smoothing and is specific to SN/VTA with no effect in other decision or control areas (*SI Appendix*, Fig. S2).

### A Computational Mechanism for the Effect of Endogenous Fluctuations on Risk Taking.

We next examined how endogenous fluctuations in SN/VTA BOLD activity influenced risk taking and tested whether the effect was specific to a certain set of offers. We computed the difference between the average return for risky and safe options and identified a main effect of this value difference, indicating that increased value for risky relative to safe options was associated with an increased propensity to choose the risky option [*F*_(2.822,__118.505)_ = 107.580, *P* < 0.001; Fig. 2*C*]. We found a main effect of endogenous fluctuations in SN/VTA activity on risk taking [*F*_(1,42)_ = 14.356, *P* < 0.001; Fig. 2*C*] but no interaction with value difference [*F*_(3.113,__130.749)_ = 0.127, *P* = 0.95], indicating that low SN/VTA activity is associated with greater risk taking irrespective of how much risky and safe options differed in value. We also found no interaction between risky option value (i.e., £6, £9, or £12) and activity [*F*_(1.957,__82.208)_ = 0.493, *P* = 0.61], further supporting an association between low endogenous SN/VTA activity and a value-independent increase in risk taking.

We next asked whether endogenous SN/VTA BOLD activity influenced option valuation in a manner consistent with standard economic models. Model comparison revealed that a parametric model based on prospect theory (19) provided a good description of behavior (pseudo-*R*^2^ = 0.44 ± 0.15) but was outperformed by a model (20, 21) that included a gambling bias parameter (pseudo-*R*^2^ = 0.55 ± 0.12; *Methods* and *SI Appendix*, Table S1). Changes in this gambling bias parameter *κ* shift the sigmoidal decision function in standard models, capturing a propensity to take risks irrespective of offer value. This gambling bias parameter was significantly higher in low compared to high activity conditions [*t*_(30)_ = 2.21, *P* = 0.04]. No differences were observed for other model parameters [risk aversion *α*: *t*_(30)_ = −0.5, *P* = 0.62; inverse temperature *µ*: *t*_(30)_ = 0.13, *P* = 0.9; Fig. 2*D*]. This finding suggests that endogenous SN/VTA activity does not impact the valuation process in a value-dependent way, but instead influences a more general decision process that does not depend on the relative values of available options.

Variability (i.e., SD) in SN/VTA BOLD activity was uncorrelated with the difference in risk taking between low and high activity across subjects (Spearman *ρ* = −0.19, *P* = 0.29). By design, all subjects were offered a set of options in the real-time fMRI task such that each should gamble one-half of the time on average. However, the percentage of risky choices was negatively correlated with the difference in risk taking between low and high SN/VTA activity (Spearman *ρ* = −0.46, *P* = 0.002). This means that the decisions of people who gamble less than predicted by prospect theory are more susceptible to endogenous SN/VTA fluctuations.

Dopamine activity is known to influence behavior in multiple ways. For example, high tonic dopamine is proposed to mediate an enhanced motivational vigor (22, 23), leading to faster reaction times. We reasoned that if endogenous SN/VTA BOLD fluctuations reflect changes in tonic dopamine, subjects should choose more quickly when endogenous activity is high. Matching this prediction, we found faster reaction times in high (1.67 ± 0.05 s) compared to low (1.72 ± 0.05 s) activity conditions [*t*_(42)_ = 3.13, *P* = 0.003; *SI Appendix*, Fig. S3], consistent with an influence of tonic dopamine on endogenous SN/VTA BOLD activity. We conducted an additional multiple linear regression and predicted reaction times based on SN/VTA BOLD activity, choice to safe or risky option, and the absolute value of the difference in option subjective values, an index of choice difficulty. Even when controlling for these variables, reaction times were still significantly related to SN/VTA BOLD activity [*t*_(42)_ = 3.08, *P* = 0.004].

### Endogenous Fluctuations Affect Phasic Responses During Choice.

We next asked how endogenous SN/VTA BOLD fluctuations shifts preferences in risky decision making as described in our computational model. Given a known association between baseline activity and task-evoked responses (4), we hypothesized that endogenous SN/VTA BOLD fluctuations impact risk taking through an influence on the expression of phasic task-evoked activity known to represent choice-relevant information (14).

**Fig. 3.**
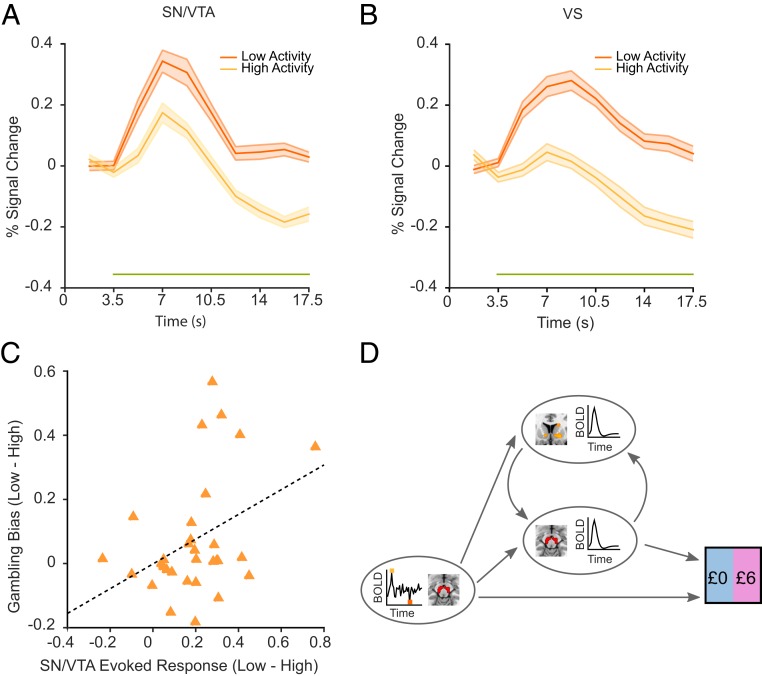
Endogenous fluctuations modulate risk taking via task-evoked responses. (*A* and *B*) Endogenous fluctuations lead to distinct task-evoked response patterns with greater BOLD responses in SN/VTA and VS when offers are presented against a background of low endogenous SN/VTA activity. Percent signal change was calculated relative to the 2 volumes following stimulus onset to correct for differences in starting baseline. The green horizontal line indicates statistical significance (*P* < 0.01). (*C*) The effect of endogenous SN/VTA activity on risk taking is associated with phasic task-evoked SN/VTA responses. Subjects with a larger difference in task-evoked responses between low and high activity conditions had larger differences in gambling bias parameter *κ* (*r* = 0.39; *P* = 0.03). (*D*) Mediation analysis shows task-evoked VS responses mediate the influence of endogenous SN/VTA fluctuations on risk taking through their influence on task-evoked SN/VTA responses, indicating the effect of endogenous SN/VTA fluctuations on behavior is under the influence of reciprocal dynamics between SN/VTA and VS.

We examined task-evoked SN/VTA responses and found that phasic responses to offer presentation were significantly increased in low compared to high prestimulus activity (Fig. 3*A*; *P* < 0.01, cluster-extent permutation test, height threshold *t* = 2, 5,000 permutations). We next examined task-evoked responses in ventral striatum (VS) and ventromedial prefrontal cortex (vmPFC), regions that receive dense dopaminergic innervation (24, 25) and express strong functional connectivity with the SN/VTA (*SI Appendix*, Fig. S4). We found the same effect as in SN/VTA with low endogenous SN/VTA BOLD activity leading to larger phasic task-evoked responses in both VS (*P* < 0.01, cluster-extent permutation; Fig. 3*B*) and vmPFC (*P* < 0.01; *SI Appendix*, Fig. S5*A*). Consistent with previous studies (26), we also found that phasic BOLD responses in VS and vmPFC reflected the subjective values of options (*SI Appendix*, Fig. S6).

We reasoned that if task-evoked responses play a critical role in translating endogenous fluctuations into risky choice, then subjects with stronger effects of endogenous SN/VTA BOLD fluctuations on task-evoked responses should show a greater difference in risk taking in low compared to high activity conditions. We found this to be the case, with larger effects of endogenous SN/VTA fluctuations on task-evoked SN/VTA responses predicting larger increases in the gambling bias parameter *κ* (*r* = 0.39, *P* = 0.03; Fig. 3*C*). This effect was specific to phasic SN/VTA responses as there was no such effect in decision and control areas (all *P* > 0.1).

Last, we investigated the relative contributions of both endogenous SN/VTA BOLD fluctuations and task-evoked responses to risk taking using multilevel mediation analyses (27). We found that SN/VTA BOLD activity significantly impacted task-evoked response in both SN/VTA and VS (Fig. 3*D* and *SI Appendix*, Fig. S5 and Table S2). Task-evoked SN/VTA responses modulated risk taking, but task-evoked VS responses influenced risk taking only indirectly through their impact on task-evoked SN/VTA responses (*SI Appendix*, Fig. S5). These results show that endogenous SN/VTA BOLD fluctuations shape decision making through their influence on task-evoked responses to offers in a decision network.

## Discussion

The brain expresses substantial ongoing activity in the absence of external stimulation. Although many studies capitalize on this fact and have described this “resting state” (2), little is known about the function of spontaneous fluctuations and whether it carries relevance for higher-order cognition. We show that endogenous fluctuations in the dopaminergic midbrain have direct behavioral relevance in modulating a preference for risky decision making in humans. Using a framework to study the influence of intrinsic fluctuations on behavior, we find greater risk taking when choice options are presented against a background of low compared to high SN/VTA BOLD activity. Our findings highlight that the endogenous state of a network relevant for behavior is critical for determining which actions are taken.

We show that endogenous SN/VTA BOLD activity influences risky decisions via modulation of phasic task-evoked responses to potential rewards. Our results are consistent with findings that impulsive behavior is linked to phasic dopamine release (13, 28) and to levodopa administration (11, 12), assumed to increase phasic dopamine (29). Low prestimulus activity and levodopa administration may both exert their effects on risk taking by boosting task-evoked phasic responses, which in turn promote risk-taking behavior.

Reward-predicting cues elicit phasic responses in midbrain dopamine neurons (30). In rodents, optogenetic manipulation of SN dopamine neurons boosts striatal dopamine release and biases action selection (31). Optogenetic stimulation of striatal D2-receptor neurons modulates risk preferences (32). Attenuation of prechoice phasic dopamine via electrical stimulation of the lateral habenula reduces preference for risk in rodents (33). Our study builds on these results by identifying a possible link between prestimulus brain activity, phasic responses to stimuli, and subsequent risky choice.

The functional role of these endogenous fluctuations remains unclear, but they might form a reference point relative to which potential offers are evaluated. While standard models of economic decision making often treat preferences as independent of a rational agent’s current state, real-world behavior often reflects comparison against a reference point that can change over time (34), sometimes substantially changing the subjective value of an offer (35). If endogenous SN/VTA BOLD activity reflects slow dopaminergic fluctuations, proposed to index environmental reward rate (22, 23) or reward anticipation (36), then these fluctuations could represent a reference point against which potential rewards are compared during decision making (37, 38). Potential rewards presented on a background of low activity could lead to enhanced task-evoked responses linked to greater risk taking (33).

Endogenous fluctuations may constitute an evolutionarily conserved principle that enables the brain to introduce variability across a wide variety of processes including perception (5, 6) and motor action (7). Neural variability has been hypothesized to reflect the dynamic range of potential responses to environmental stimuli, allowing the brain to flexibly transition between states in response to changing task demands (39). It could also reduce susceptibility to becoming entrenched in specific behavioral repertoires (40) and promote exploration in dynamic environments that are a common feature of the natural world (41).

One possible source of variability that could relate to our results is D2/D3 autoreceptor availability in the SN/VTA. Lower autoreceptor availability is associated with greater dopamine release following amphetamine administration and greater trait impulsivity (13). The link we find between risk taking and phasic responses is also consistent with the finding that phasic dopamine during gambling tasks is greater in pathological gamblers (28).

One limitation of our study is that BOLD activity is an indirect measure of local neuronal activity thought to consist of an ensemble of signals including afferent and recurrent inputs (42). Phasic and tonic dopamine release may contribute to fluctuations in SN/VTA BOLD activity, while optogenetic stimulation of dopamine neurons in VTA is sufficient to elicit BOLD activity in VTA (43). However, SN/VTA BOLD activity may also reflect activity in other cell types including glutamatergic (44) and GABAergic neurons that act to inhibit dopamine neurons when reward is expected (45). Reduced GABAergic activity could also be associated with greater phasic dopamine release and provide an alternate explanation for greater risk taking when prestimulus SN/VTA BOLD activity is low.

Previous studies have shown that dopamine release in VS, as measured using positron emission tomography, is linked to reward-related SN/VTA BOLD activity (46). While we have focused on the SN/VTA, the extent to which BOLD activity in the downstream VS responds to a cue is tightly coupled to SN/VTA BOLD activity. Future studies might extend these findings with direct striatal recordings that assess the relationship between spontaneous fluctuations and risk taking.

Our effect is consistent across individuals, albeit modest in terms of effect size (on average, a 3.4% increase in the number of risky options chosen). We would predict a larger effect size with direct electrophysiological recordings, since fMRI measurements are inherently noisy at several levels (47). However, given the many factors that contribute to risky decision making, it would be surprising if the state of the brain when options are presented had a large effect on the probability of risky decisions, especially in the absence of any environmental changes.

The effect size is comparable in size to previous studies. For example, a standard clinical dose of 150 mg of levodopa increased risk taking by only 5% on average (12) and natural aging leads to a comparable decrease in risk taking, which we surmised may reflect age-related dopaminergic decline estimated at 5–10% per decade (48). It is also noteworthy that the effect of low prestimulus SN/VTA activity on risk taking is particularly large in relative terms for unattractive gambles. The probability that individuals choose the least attractive gambles (chosen less than 20% of the time) is much greater under low than high prestimulus activity (18.7% vs. 15.4%), a 21% relative increase. In contrast, the probability that individuals choose the most attractive gambles (chosen more than 80% of the time) is only 5% greater under low than high prestimulus activity in relative terms. Our findings may be particularly relevant to understanding pathological gamblers, who may take risks that others would generally avoid.

Our key finding is that variability in higher-order cognition can emerge out of a neurophysiologically well-defined process. While risk preferences are thought of as personality traits determined partly by genetic variation (49), we show that the expression of risk preferences reflects in part individual susceptibility to endogenous fluctuations. Neural variability may change with task experience, consistent with reductions in neural variability during skill learning (50) and the impact of endogenous fluctuations may be largest in novel environments. Aberrant endogenous fluctuations might also play a role in disorders where there is excessive behavioral variability or risk taking, such as attention deficit hyperactivity disorder (51) and pathological gambling (28). Accounting for the influence of endogenous neural fluctuations on behavior is critical for understanding the neurobiological processes underlying cognition in health and disorder.

## Methods

### Subjects.

Forty-nine healthy, young adults (age 25.2 ± 4.2; mean ± SD) were recruited through the University College London (UCL) Psychology Subject Database. Subjects were screened to ensure no history of neurological or psychiatric disorders. Six subjects were excluded from analyses: 3 subjects because of excessive number of missed trials (>20) and 3 due to frequent large head movements (>3 mm). A total of 43 subjects (group 1: 10 females, 2 males; group 2: 21 females, 10 males) were included. Subjects in both groups went through identical procedures with the only difference being that the range of values for the safe options, drawn around each subject’s indifference points, was wider for group 2 than group 1, allowing us to better distinguish between competing computational models (*Procedure*). The study was approved by the UCL research ethics committee, and all subjects gave written informed consent.

### Procedure.

Our study protocol spanned 2 sessions ∼24 h apart. On the first day, we assessed gambling behavior and collected structural brain scans. These scans were used to define individualized anatomical masks of the dopaminergic midbrain for use in the following session. On the second day, decision making was reassessed before subjects participated in the real-time fMRI experiment reported.

#### Day 1.

##### Probabilistic gambling task.

Subjects first played a probabilistic gambling task consisting of 180 trials. On each trial, subjects chose between a certain monetary amount and a gamble with equal probabilities of 2 outcomes. There were 3 gamble options available: £0 and £6, £9, or £12. The certain amounts were determined using 12 divisors (0.82, 0.87, 0.93, 1, 1.1, 1.23, 1.4, 1.6, 1.9, 2.25, 2.75, and 3.5) on the expected value of the gambles, chosen to accommodate a wide range of risk sensitivity. Take, for example, a fraction of 3.5 and a gamble between £0 and £6. The expected value of a £0 or £6 gamble is £3 (0.5 × £0 + 0.5 × £6), which divided by 3.5 gives a certain amount of £0.86. There were 12 certain amounts for each gamble option in total, and each trial was repeated 5 times in a randomized sequence.

##### Structural scans.

Multiparameter maps were acquired for each subject (52). The magnetization-transfer (MT) saturation image was used for the drawing of the region of interest (ROI) (SN/VTA) due to its ability to delineate gray and white matter in subcortical/brainstem regions, in line with preceding studies (16, 53).

#### Day 2.

Prior to the real-time fMRI session, subjects completed a shorter version of the probabilistic gambling task consisting of 108 trials to recalibrate the subjects’ indifference points. The only difference between this task and the task on day 1 was that each trial was repeated 3 instead of 5 times.

##### Probabilistic gambling task inside the MRI scanner.

Choice behavior across both days was fitted to a prospect theory-based parametric decision model that has been used in past studies (12, 19) to describe decision making under risk. The expected utility of the certain options and gambles were determined using the following equations:

$$U_{\text{gamble}}=0.5{\left(V_{\text{gain}}\right)}^{\alpha },$$

$$U_{\text{certain}}={\left(V_{\text{certain}}\right)}^{\alpha },$$

where *V*_gain_ is the value of the potential gain from a gamble and *V*_certain_ is the value of the certain option. *α* alters the degree of curvature of the utility function and represents the degree of risk aversion. When presented with an option where the expected values for the certain gain and the gamble are equal, a subject with *α* = 1 would be risk-neutral and indifferent between the 2, a risk-seeking individual with *α* > 1 would choose the gamble more often, and a risk-averse individual with *α* < 1 would choose the certain gain more often. The probability of selecting a gamble was determined by the following softmax rule:

$$P_{\text{gamble}}=\frac{1}{1+e^{-µ\left(U\mathrm{gamble}-U\mathrm{certain}\right)}}\;,$$

where the degree of stochasticity in choice behavior is captured by the inverse temperature parameter *μ*. When *μ* is low, subjects are more likely to choose randomly between safe and risky options irrespective of their subjective values. When *μ* is high, subjects increasingly choose the action leading to the highest expected reward. Expected utilities for the certain option were sampled evenly (5 bins) between *P*_gamble_ = 0.3 and 0.7 for each gamble level for the first group of subjects, and *P*_gamble_ = 0.1 and 0.9 for the second group of subjects. These utilities were then converted back to objective values and used as the safe options in the real-time fMRI session. The real-time fMRI task consisted of 90 trials in total with 30 trials for each gamble level (£0 and £6, £9, or £12) of which 15 trials were allocated to the low baseline condition and 15 trials allocated to the high baseline condition according to criteria defined in the following section.

### Real-Time fMRI.

#### Software and preprocessing of images.

Real-time preprocessing of the functional data was performed using Turbo-BrainVoyager (TBV) (Brain Innovation) and custom scripts. Time courses for every voxel within the SN/VTA ROI were extracted from smoothed and realigned images (6-mm full width at half-maximum) and exported using TBV. Exported data were then corrected for additional noise sources (movement and physiological noise; see below). Physiological noise arising from breathing and pulsatile artifacts (*SI Appendix*) were incrementally regressed out in real time from the exported time courses using a custom-made MATLAB (MathWorks) toolbox. The ensuing filtered time courses were then analyzed to detect endogenous fluctuations.

#### Quantifying the level of BOLD activity.

We used a sliding window approach to quantify endogenous activation of the SN/VTA over the course of the experiment. This measure not only takes scanner-induced and other slow signal drifts (e.g., due to a warming of the gradient coils) into consideration but is also robust to outlier activations and can account for changes in the variance of the signal over time. A normal cumulative distribution function was used to quantify the distribution of BOLD signal within an ongoing sliding window consisting of 69 volumes (∼2 min). The mean of the most recent 2 volumes was compared to the previous 69 volumes over the progression of the entire experiment. The distribution of the sliding window was updated with each new volume acquired. Thresholds for the trials were set below the 15th percentile for low baseline trials and above the 85th percentile for high baseline trials. When BOLD activity exceeded the thresholds, a trial was immediately presented. There was a minimum intertrial interval of 20 s to allow the hemodynamic response for each trial to return close to baseline. If threshold criteria were not met by 55 s, a trial was presented and categorized as low or high depending on whether it was lower or higher than the mean of the preceding baseline, respectively. This procedure was applied to the 15.1 ± 5.8% (mean ± SD) of trials that did not reach the threshold criteria.

#### Image acquisition.

MRI data were acquired at the Wellcome Centre for Human Neuroimaging at UCL, using a Siemens Trio 3-tesla scanner equipped with a 32-channel head coil. A partial-volume 2D echo-planar imaging (EPI) sequence that was optimized for striatal, medial prefrontal, and brainstem regions was selected for the functional images. Each volume consisted of 25 slices with 2.5-mm isotropic voxels (repetition time, 1.75 s; echo time, 30 ms; slice tilt, −30°). At the beginning of each functional session, 10 EPI volumes were acquired with the 10th volume selected as the template used to coregister the ROI. Field maps with 3-mm isotropic voxels (whole-brain coverage) were also acquired to correct the EPIs for any inhomogeneity in magnetic field strength. Subsequently, the first 6 volumes of each run were discarded to allow for T1 saturation effects. Sequence settings were identical across subjects (e.g., no variation in tilt angle) and no slices were discarded. Overlapping coverage across all subjects is indicated in Fig. 1. Structural images were also acquired for all subjects (see *SI Appendix* for full details).

#### ROI definition and transformation.

Bright areas in MT-contrast images have been shown to be coextensive with the SN as delineated histologically by tyrosine hydroxylase immunohistochemistry, which stains dopaminergic neurons (54) that are the key component of SN/VTA. Leveraging upon this, SN/VTA ROIs were hand-drawn for each individual in MRIcron (55) using MT-weighted structural images. In accordance with procedures outlined previously (56), medial and lateral boundaries of the SN/VTA ROI were defined based on the change in contrast between its bright gray color and the dark gray color of the adjacent cerebral peduncle and interpeduncular fossa. Lower and upper boundaries of the ROI were selected as the slices preceding the ones where the intensity of SN/VTA was indistinguishable from surrounding tissue, totaling between 6 and 9 slices contingent on individual SN/VTA size differences. To prepare the hand-drawn SN/VTA ROI for use in TBV, it needs to be coregistered and transformed to the space and resolution of the EPIs. Coregistration was carried out using a single EPI volume as the reference image, and the individual-specific T1-weighted image as the source image. Following this, the EPI voxels corresponding to each ROI voxel were indexed based on Euclidean distance calculated in native space. Since the coordinate space in TBV differs from more common ones such as the Montreal Neurological Institute (MNI) space, coordinates for the ROI were transformed before use in TBV. This series of coregistration and transformations was executed using custom MATLAB scripts available on GitHub (https://github.com/tuhauser/rtfMRI).

### Off-Line Analyses.

Images were preprocessed using standard procedures in SPM 12 (Wellcome Centre for Human Neuroimaging, UCL; *SI Appendix*). The mediation analysis (*SI Appendix*) presented here tests whether baseline SN/VTA BOLD activity influences the magnitude of task-evoked responses in SN/VTA [VS/vmPFC] (path a), whether task-evoked responses in SN/VTA [VS/vmPFC] are correlated with choice controlling for SN/VTA baseline (path b), whether the relationship between SN/VTA baseline and risk taking is reduced after controlling for task-evoked responses (path c′), and finally a test of mediation. A mediator can be interpreted as an indirect pathway through a brain region that links endogenous fluctuations in SN/VTA baseline activity with choice, whereby this relationship would be reduced or abolished if the mediator is disrupted. To further understand how task-evoked responses in VS mediates baseline SN/VTA BOLD activity and choice despite the absence of a direct link between VS and choice, we conducted an additional analysis using task-evoked responses in VS to predict choice using task-evoked responses in SN/VTA as a mediating variable.

### Computational Modeling.

#### Parametric decision model based on prospect theory.

Details of this model are provided above (Procedure, Day 2). This model provided a good fit for choice behavior in both low- and high-activity conditions with an average pseudo-*R*^2^ of 0.44 (SD, 0.15). For the real-time fMRI analysis, we compared this standard model to 3 alternative models including the one below and conducted a model comparison.

#### Parametric decision model based on prospect theory with gambling bias.

To account for the possibility of a shift in indifference points leading to a difference in tendencies to choose gambles, the softmax rule in the parametric prospect theory model included an additional parameter, *κ*, such that:

$$P_{\text{gamble}}=\frac{1}{1+e^{-µ\left(U\mathrm{gamble}-U\mathrm{certain}+\kappa \right)}}.$$

*κ* here represents a gambling bias that is additive to the expected utilities. This model provided the best fit out of all of the models tested with a pseudo-*R*^2^ of 0.55 (SD, 0.12). Model comparison based on Bayesian information criterion confirmed the fit and revealed that this model fitted the data best (see *SI Appendix*, *SI Methods* for alternative models, and *SI Appendix*, Table S1). Larger effects of endogenous SN/VTA fluctuations on task-evoked SN/VTA responses (as measured using the average of an epoch corresponding to 5.25 to 10.5 s in Fig. 3*A*) correlated with larger increases in gambling bias parameter.

## Supplementary Material

Supplementary File
