# Supplementary material for: Endogenous fluctuations in the dopaminergic midbrain drive behavioral choice variability
Source: Proc Natl Acad Sci U S A. 2019 Aug 26;116(37):18732–7. doi: 10.1073/pnas.1900872116 (PMC6744888; doi:10.1073/pnas.1900872116)
Supplement: Supplementary File [file pnas.1900872116.sapp.pdf]

PNAS
www.pnas.org

Supplementary Information for

Endogenous Fluctuations in the Dopaminergic Midbrain Drive Behavioral Choice Variability

Benjamin Chew*, Tobias U. Hauser*, Marina Papoutsi, Joerg Magerkurth, Raymond J. Dolan, and Robb B. Rutledge

Benjamin Chew, Tobias U. Hauser
Email: benjamin.chew.13@ucl.ac.uk, t.hauser@ucl.ac.uk

This PDF file includes:

Figs. S1 to S6
Tables S1 and S2

www.pnas.org/cgi/doi/10.1073/pnas.1900872116

SI Methods

Real-time fMRI

Physiological Noise. To remove physiological noise arising from breathing and pulsatile artifacts, subjects were fitted with a pneumatic respiratory belt and a pulse oximeter. Physiological measurements from these devices were modeled using a Fourier expansion of physiological phases based on the RETROICOR model[1] and respiratory volume[2]. These were incrementally regressed out in real time from the exported time courses using a custom-made MATLAB (MathWorks, Natick, USA) toolbox. The ensuing filtered time courses were then used in the main experiment.

fMRI Image Acquisition

MRI was acquired at the Wellcome Centre for Human Neuroimaging, UCL, using a Siemens Trio 3-Tesla scanner equipped with a 32-channel head coil. A partial-volume 2D echo-planar imaging (EPI) sequence that was optimized for striatal, medial prefrontal, and brainstem regions was selected for the functional images. Each volume consisted of 25 slices with 2.5mm isotropic voxels (repetition time (TR): 1.75s; echo time (TE): 30ms; slice tilt: -30°). At the beginning of each functional session, 10 EPI volumes were acquired with the 10th volume selected as the template used to co-register the ROI. In addition, field maps with 3mm isotropic voxels (whole brain coverage) were also acquired to correct the EPIs for any inhomogeneity in magnetic field strength. Subsequently, the first 6 volumes of each run were discarded to allow for T1 saturation effects. Structural images consisted of 3 spoiled multi-echo 3D fast low angle shot (FLASH) acquisitions at 0.8mm isotropic resolution with T1 (TR: 18.7ms; flip angle: 20°), proton density (PD) (TR: 23.7ms; flip angle: 6°), and magnetization transfer (MT) (TR: 23.7ms; flip angle: 6°; excitation preceded by a 2kHz off-resonance Gaussian radiofrequency (RF) pulse with 4ms duration and 200° nominal flip angle) weightings. Additional B1 mapping and field maps were acquired to get calibration data measuring the spatial distribution of the B1+ transmit field in order to detect the spatial variation in flip angle. Sequence settings were identical across subjects (e.g., no variation in tilt angle) and no slices were discarded. Overlapping coverage across all subjects is indicated in Figure 1.

fMRI Offline Analyses

Images were preprocessed using standard procedures in SPM 12 (Wellcome Centre for Human Neuroimaging, UCL). This consisted of unwarping EPIs using field maps, motion correction, spatial transformation to the MNI template, and spatial smoothing with a 6-mm full-width at half-maximum Gaussian kernel.

Multilevel mediation analysis was carried out using the Mediation Toolbox (http://wagerlab.colorado.edu/tools)[3]. For the mediation analysis, evoked responses in the SN/VTA, VS, and vmPFC were determined as the maximum percentage change in BOLD signal within a 10s epoch following trial onset, while baseline SN/VTA was determined as

the percentile that each trial was triggered off (see previous section). Distribution of path coefficients were estimated by drawing 10,000 random samples and significance estimates were computed through bootstrapping.

Computational Modeling

Parametric Approach-Avoidance Decision Model. A recent model[4] that was developed to account for value-independent tendencies to choose gambles is the approach-avoidance model, which allows choice probabilities to differ from 0 or 1 in the limit when a softmax rule is used. Expected utilities were determined using equations in the prospect theory model described earlier.

The main difference lies in the softmax rule where the probability of gambling depended on a new parameter, β, determined by the following equations:

$$P_{gamble} = \frac{1-\beta}{1+e^{-\mu(Ugamble-Ucertain)}} + \beta \text{ if } \beta \geq 0$$

$$P_{gamble} = \frac{1+\beta}{1+e^{-\mu(Ugamble-Ucertain)}} \text{ if } \beta < 0$$

If β is positive, choice probabilities are mapped from (β, 1). If β is negative, choice probabilities are mapped from (0, 1+β). This model provided a good fit of behavior with an average pseudo-R^2 of 0.47 (SD: 0.14).

Parametric Decision Model Using Expected Values. The final model tested was one that used the expected values of the gamble (E_{gamble}) and certain gain ($E_{certain}$) and passed through the following softmax with the same gambling bias term β as before:

$$P_{gamble} = \frac{1}{1+e^{-\mu(Egamble-Ecertain+\kappa)}}$$

This model had the lowest fit with a pseudo-R^2 of 0.36 (SD: 0.17), which suggests that more of the variance could be accounted for by the inclusion of a risk aversion parameter to convert objective values into subjective values.

Control Analysis

To validate the results obtained from our online procedure and to examine whether the effect of endogenous BOLD activity on risky choice behavior was a general property across the brain, we sampled activity from multiple regions. The ROI for vmPFC was derived from www.neurosynth.org, the VS ROI was bilateral 8-mm spheres at MNI coordinates derived from a previous study[5], a group anatomical mask from a previous study[6] was used for SN/VTA ROI, and the primary auditory cortex (A1) was Brodmann Areas 41 from the Wake Forest University PickAtlas toolbox for SPM[7].

BOLD time courses for these ROIs were extracted and filtered using an incremental GLM with the same motion and physiological regressors as in the real-time fMRI experiment. Based on our real-time procedure, BOLD activity for each region was averaged for the 2 most recent TRs prior to trial presentation and compared against each preceding baseline window of 2 minutes. As our design was optimized to detect activity fluctuations in the SN/VTA, the threshold used to categorize trials as low or high activity in the SN/VTA would be overly conservative when applied to other brain regions. This would lead to many trials being left uncategorized. To ensure that all trials were categorized, we relaxed the threshold and categorized each trial as low or high depending on whether pre-trial BOLD activity for each of these regions was lower or higher than the mean of the preceding baseline period.

To test whether our main effect of risk preference change is specific to SN/VTA BOLD activity, we investigated the relationship between endogenous fluctuations of BOLD activity in other brain regions and risky choice. We conducted offline analyses on A1 as a control area, as well as VS and vmPFC, which are regions strongly implicated in value-based decision making[8]. We used independent ROIs for all areas including SN/VTA and re-categorized trials based on endogenous activity in each of these ROIs.

Risky choice behavior was significantly greater for low compared to high baseline activity in the independent SN/VTA ROI (low baseline activity: 59.7 ± 1.5% (mean ± SEM), high baseline activity: 56.0 ± 1.9%, t_{42} =2.92, P = 0.003). There was no significant relationship between risk taking and endogenous activity in any of the control and decision-related areas tested (Fig. S2A). Risk taking was similar for low and high baseline activity in VS (low: 58.6 ± 1.7%, high: 57.0 ± 1.8%, t_{42} = 0.95, P = 0.35), vmPFC (low: 58.6 ± 1.6%, high: 57.1 ± 1.9%, t_{42} = 1.01, P = 0.32), and A1 (low: 58.9 ± 1.6%, high: 57.0 ± 1.8%, t_{42} =1.43, P = 0.16). To further verify that the effects we observe are driven by local rather than global fluctuations, we tested whether SN/VTA activity was still predictive of risk taking even after controlling for activity in control area A1 (t_{42} = 2.34, P = 0.02). These findings suggest that the effect is not a general effect of low and high BOLD activity modes across the brain, but specific to local fluctuations in the dopaminergic midbrain that explain variability in risk taking.

A caveat of the above analysis is that the absence of any effect in a control area could be due to reduced endogenous signal variability. To rule out this alternative explanation, we calculated the signal change of epochs used to trigger each trial relative to their preceding baselines. Differences in signal change between low and high activity conditions were largest in vmPFC and smallest in VS, suggesting that activity used to trigger trials in SN/VTA was no more extreme than that observed in other regions, supporting our finding of a specific effect of SN/VTA endogenous fluctuations on risk taking (Fig. S2B).

As the VS results may be affected by partial volume effects due to its location and the image acquisition parameters, we re-ran the preprocessing steps and re-analyzed the data after discarding the top and bottom slices of the partial volumes. We found that risk taking was still similar for low and high baseline activity in VS (low: 58.7 ± 1.7%, high: 57.0 ± 1.9%, t_{42} = 1.02, P = 0.32), suggesting that the absence of an association between VS BOLD activity and risk taking was not due to partial volume effects.

As SN/VTA BOLD signals recorded in real-time may be contaminated by signals from surrounding structures due to smoothing, we also performed offline analyses on unsmoothed

functional images using the same algorithm to reclassify pre-stimulus activity and found consistent results in unsmoothed data. Risk taking was higher for trials presented against a background of low compared to high SN/VTA BOLD activity (low activity: 59.9 ± 1.8%, high activity: 55.6 ± 2.1%, t_{42} = 3.2, P = 0.003).

To test how sensitive the effect we observe is to the timing of pre-stimulus activity, we reanalysed the data, reclassifying activity levels as high or low based on volumes t-2 and t-3 before trial onset (instead of t-1 and t-2). Discarding the final volume of SN/VTA signal before trial onset did not affect the relationship between pre-stimulus activity and risk taking (t_{42} = 2.95, P = 0.005), suggesting that the effect we observe does not depend on the precise timing of option presentation.

Statistical Analysis

Descriptive and inferential statistics were carried out in MATLAB (MathWorks) with in-house scripts and functions in SPSS Statistics (IBM Corp). All behavioral analyses were conducted on trials that were matched for value between low and high activity modes. In other words, if a participant missed a trial in the low or high baseline activity condition, the corresponding trial in the other baseline condition was excluded from analyses to match the number of trials in each condition (final number of matched trials: 95 ± 6%, mean ± SD).

Precision measures (e.g., SD, SEM) are indicated in brackets where appropriate. Paired sample t-tests were used to compare reaction times and the number of risky choices between low and high activity conditions across the different brain regions tested. The main effect of gamble value bins and risk, as well as possible interactions between gamble value and endogenous activity were assessed using a repeated-measures ANOVA with Greenhouse-Geisser correction (5 gamble value bins x 2 activity conditions). Parameters in the computational models were fit separately for each condition using the fmincon function in MATLAB to minimize their negative log-likelihoods.

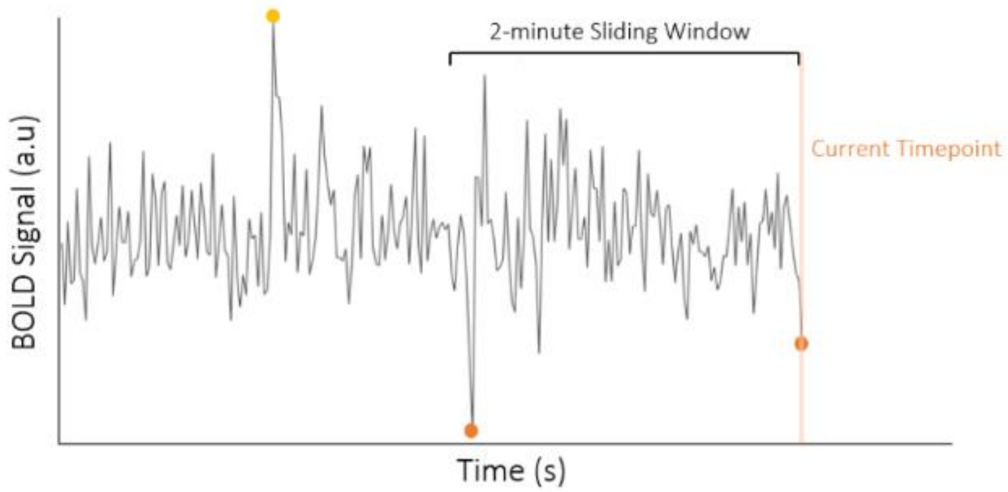

Fig S1. Task design. Endogenous activity reflecting a low/high background BOLD activity state (exceeding a 15th/85th percentile cut-off based on an incremental sliding window of 2 minutes) triggered presentation of a trial involving a choice between a safe option and a risky option. Trials presented in both low/high background activity states were matched and thus any difference in behavior can be attributed to distinct levels of endogenous SN/VTA BOLD activity.

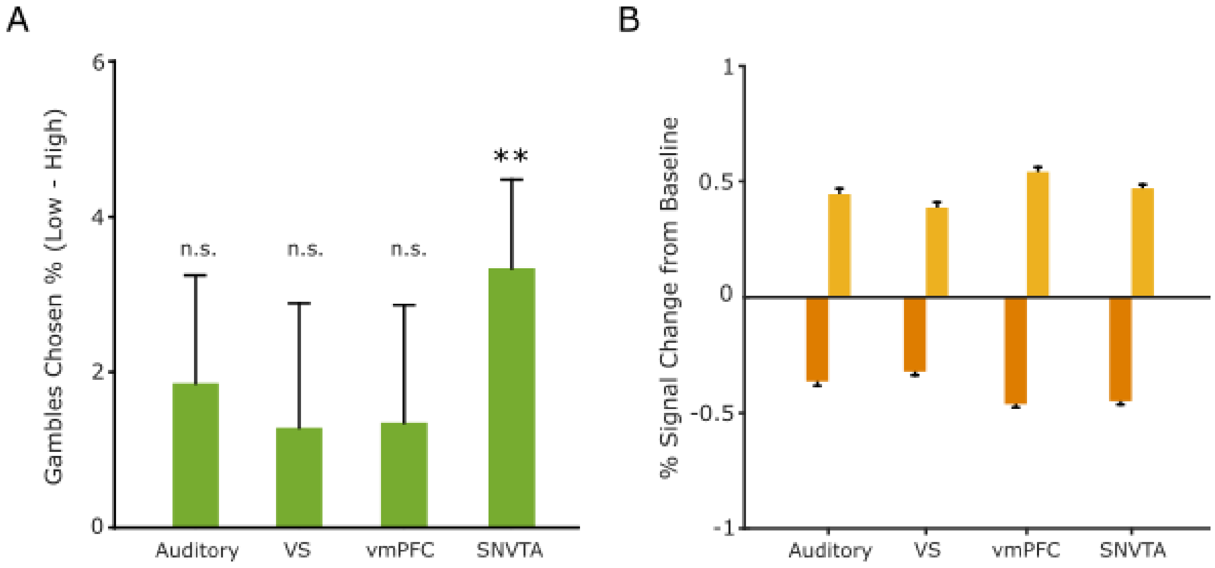

Fig S2. Control analyses. A, Offline re-classification of endogenous activity using independent ROIs (see Methods) revealed that only endogenous dopaminergic midbrain fluctuations were significantly associated with choice variability. **B,** Differences in signal change between low and high activity conditions were largest in vmPFC and smallest in VS (yellow: trials categorised as high activity, orange: signals categorised as low activity). This suggests that endogenous fluctuations in SN/VTA were not more extreme than other regions. * $P < 0.05$, ** $P < 0.01$, *** $P < 0.001$. Data are mean ± SEM.

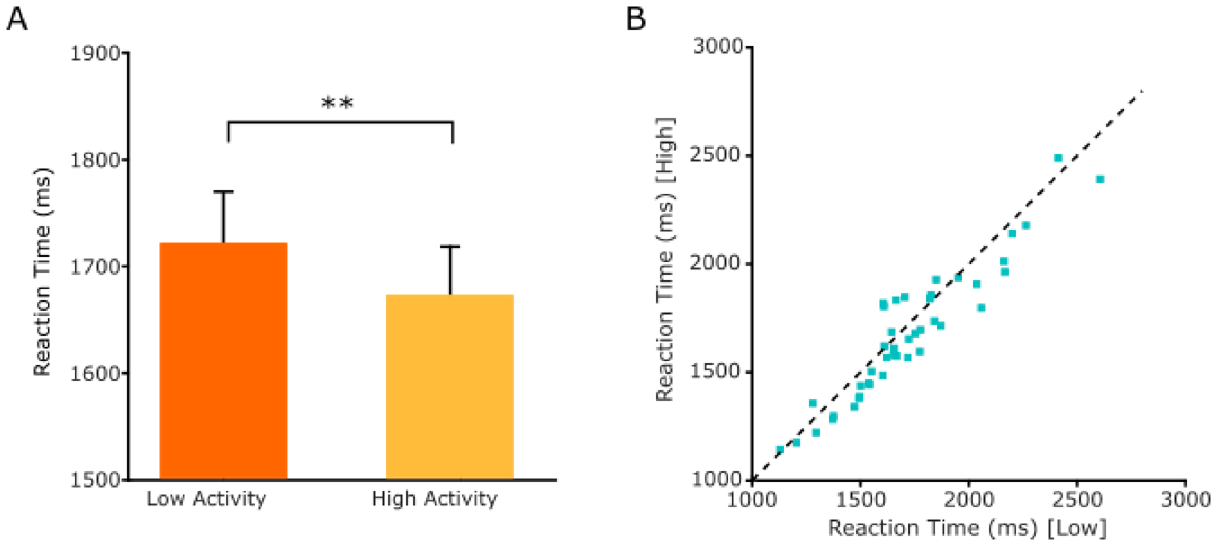

Fig. S3. Endogenous fluctuations in dopaminergic midbrain modulate vigor. A, Endogenous activity in dopaminergic midbrain modulated vigor as captured by response speed. Subjects (n = 43) were faster ($P < 0.01$) to make choices for options presented on a background of high compared to low endogenous activity. **B,** This effect of faster response speeds for high than low activity was consistent across subjects. ** $P < 0.01$.

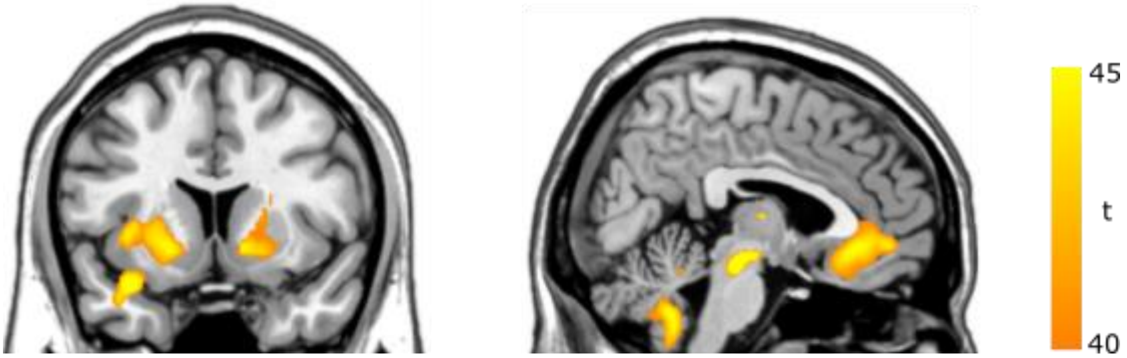

Fig. S4. Intrinsic fluctuations in dopaminergic midbrain co-activate a decision network. To identify a network of brain areas that the SN/VTA was embedded in and whose activity co-varied with endogenous fluctuations in the SN/VTA, we first extracted the BOLD time course from each subject's SN/VTA using the same independent ROI as in the offline analyses. This was then included as an additional regressor in a GLM at the 1st level analysis in SPM. T-Contrasts on this SN/VTA regressor were used in the 2nd level group analysis, revealing that activity in dopaminergic midbrain co-activates a decision-related network of areas including VS and vmPFC (both $P<0.05$, FWE-corrected).

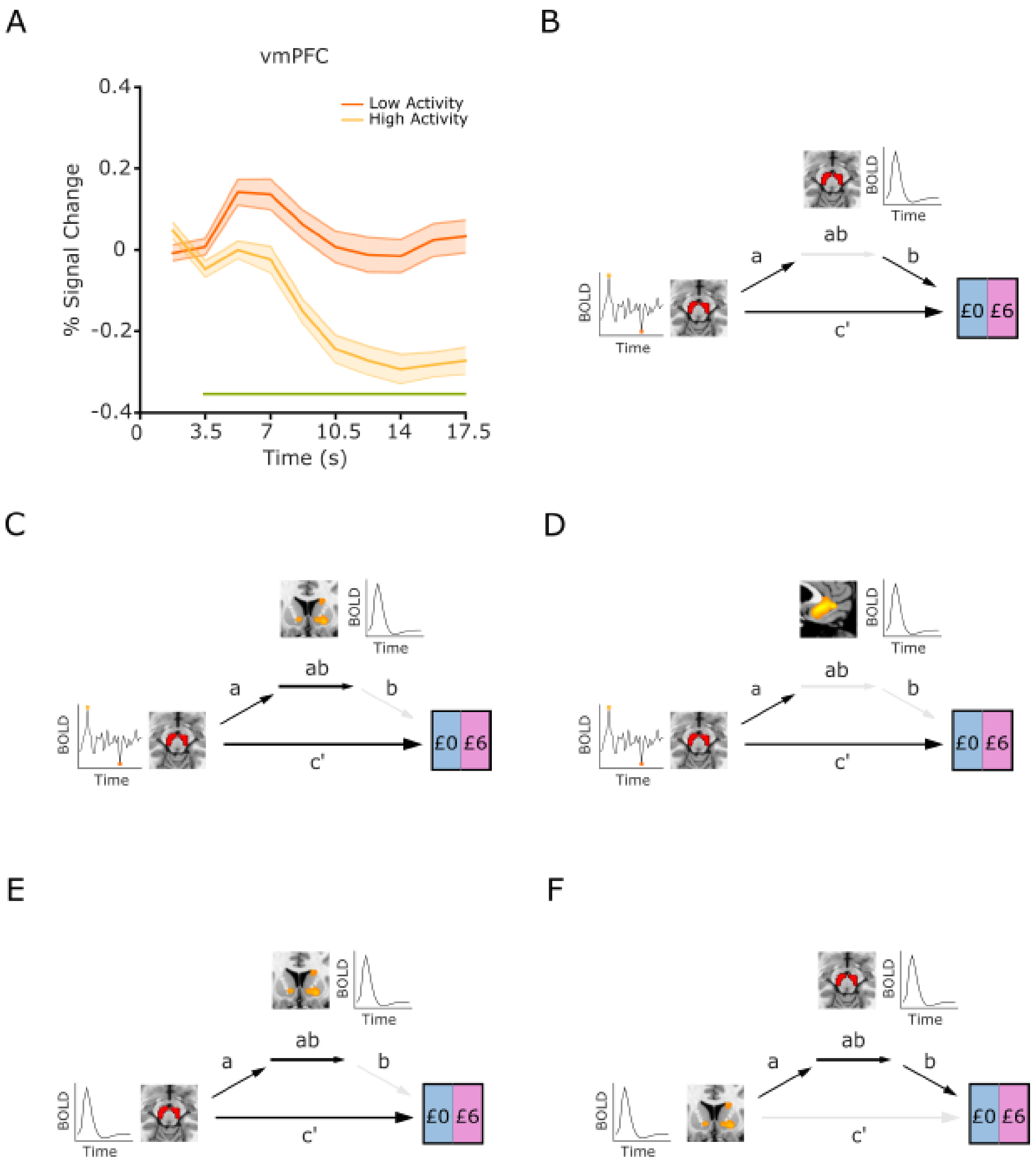

Fig. S5. Endogenous SN/VTA activity modulates task-evoked responses in a decision network. **A,** Similar to the task-evoked responses in SN/VTA and VS, vmPFC shows increased task-evoked responses on a background of low endogenous SN/VTA activity ($P < 0.01$). Multilevel mediation analyses reveal dynamic interactions in a decision network. **B,** Endogenous SN/VTA activity influences task-evoked responses in SN/VTA, which in turn modulate decision making. **C, D,** Endogenous SN/VTA activity also influences task-evoked responses in VS in **C** and vmPFC in **D**. VS thereby mediates the effect of endogenous SN/VTA activity on risk taking. **E, F,** Further analysis revealed that task-evoked responses in VS and SN/VTA dynamically interact with each other. Of these three decision areas, only SN/VTA directly influences risk taking, with task-evoked VS responses indirectly influencing behavior through their influence on task-evoked SN/VTA responses. This result indicates that endogenous SN/VTA activity induces differential phasic responses in SN/VTA and VS, which in turn interact with and dynamically influence risk taking.

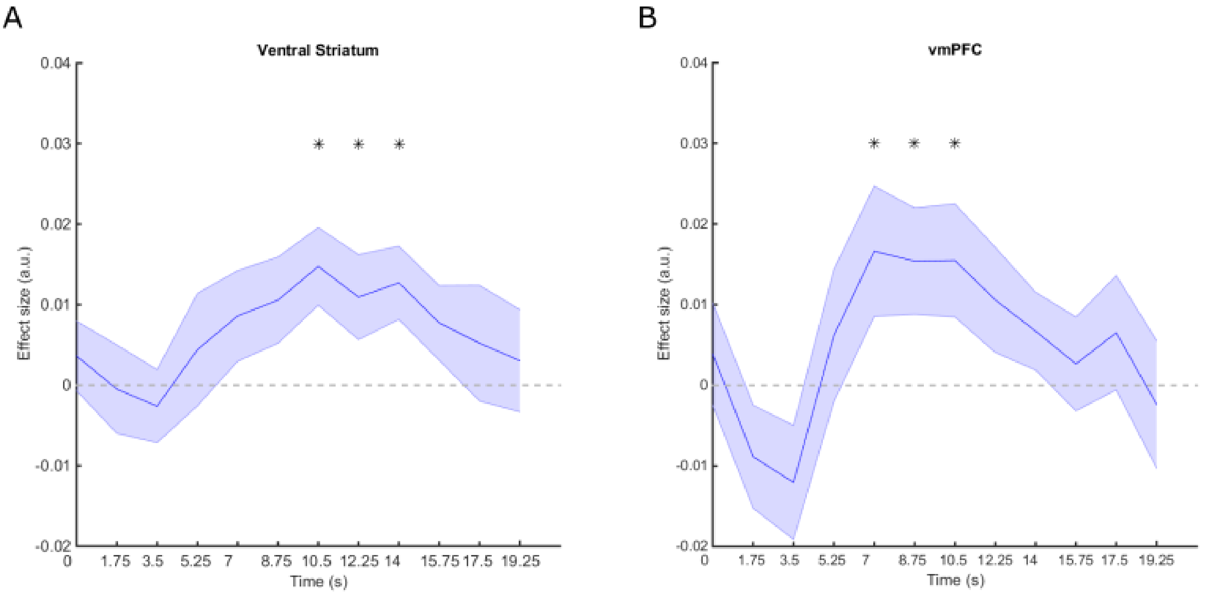

Fig. S6. Subjective value representations in VS and vmPFC. We performed a timepoint-by-timepoint analysis within VS and vmPFC ROIs derived from a meta-analysis of subjective value fMRI studies[8] and identified significant correlations between z-scored BOLD activity in both VS **(A)** and vmPFC **(B)** and the z-scored average subjective value of the two options. Choices were made at time 0. The strongest association for any timepoint was $P = 0.005$ for VS and $P = 0.026$ for vmPFC. Data are mean ± SEM. * $P < 0.05$.

Table S1. Model Comparison Results.

Model	Parameters	Mean R^2	BIC	ΔBIC
Prospect Theory	4	0.44	2636	130
Approach-Avoidance	6	0.47	2804	298
Prospect Theory with Gambling Bias	6	0.55	2506	0
Expected Values with Gambling Bias	4	0.36	2927	421

BIC measures are summed across 31 subjects. The winning model (lowest BIC) here was the parametric decision model based on prospect theory with the addition of a gambling bias. ΔBIC refers to the difference in BIC scores between each model and the winning model.

Table S2. ROI-based Mediation Results.

		SN/VTA	VS	VMPFC
Mediation of endogenous SN/VTA fluctuations on choice	Path a	-0.16*** (0.03) P < 0.001	-0.21*** (0.04) P < 0.001	-0.19*** (0.05) P < 0.001
	Path b	-0.16* (0.08) P = 0.03	-0.0011 (0.05) P = 0.98	-0.04 (0.05) P = 0.31
	Direct c’	-0.14* (0.06) P = 0.01	-0.10* (0.05) P = 0.04	-0.12* (0.05) P = 0.02
	Mediation a x b	-0.13 (0.15) P = 0.40	-0.26* (0.10) P = 0.02	0.01 (0.09) P = 0.96
Mediation of task-evoked VS responses on choice	Path a	0.52*** (0.03) P < 0.001		
	Path b	-0.24** (0.09) P = 0.006		
	Direct c’	0.13 (0.07) P = 0.08		
	Mediation a x b	-2.41** (0.89) P = 0.005		

		SN/VTA	VS	VMPFC
Mediation of task-evoked SN/VTA responses on choice	Path a		0.63***<br>(0.04)<br>P < 0.001	
	Path b		0.13<br>(0.07)<br>P = 0.07	
	Direct c’		-0.24**<br>(0.09)<br>P = 0.006	
	Mediation<br>a x b		1.58*<br>(0.78)<br>P = 0.04	

Coefficients, standard errors, and p-values for the different paths in the mediation analyses (n=43). *P < 0.05, **P < 0.01, ***P < 0.001

References

1. Glover GH, Li TQ, Ress D (2000) Image-based method for retrospective correction of physiological motion effects in fMRI: RETROICOR. *Magn Reson Med* 44(1):162–167.
2. Birn RM, Diamond JB, Smith MA, Bandettini PA (2006) Separating respiratory-variation-related fluctuations from neuronal-activity-related fluctuations in fMRI. *NeuroImage* 31(4):1536–1548.
3. Wager TD, Davidson ML, Hughes BL, Lindquist MA, Ochsner KN (2008) Prefrontal-subcortical pathways mediating successful emotion regulation. *Neuron* 59(6):1037–1050.
4. Rutledge RB, Skandali N, Dayan P, Dolan RJ (2015) Dopaminergic modulation of decision making and subjective well-being. *J Neurosci* 35(27):9811–9822.
5. Rutledge RB, Skandali N, Dayan P, Dolan RJ (2014) A computational and neural model of momentary subjective well-being. *Proc Natl Acad Sci USA* 111(33):12252–12257.
6. Hauser TU, Eldar E, Dolan RJ (2017) Separate mesocortical and mesolimbic pathways encode effort and reward learning signals. *Proc Natl Acad Sci USA* 114(35):E7395-E7404.
7. Maldjian JA, Laurienti PJ, Burdette JH (2004) Precentral gyrus discrepancy in electronic versions of the Talairach atlas. *NeuroImage* 21(1):450–455.
8. Bartra O, McGuire JT, Kable JW (2013) The valuation system: a coordinate-based meta-analysis of BOLD fMRI experiments examining neural correlates of subjective value. *NeuroImage* 76:412–427.
